# Insanity

**Published:** 1861-04

**Authors:** 


**Insanity.**

Dr. Winslow, in his views on the pathology and treatment of insanity, gives it as his opinion, that local depletion cautiously used is of great value, and that the hot bath, as practised by Dr. Brierre de Boismont, of Paris, is next in importance. For this purpose, patients are kept for eight or ten hours in water at the temperature of about 85° Fahr., with a stream of water, at the temperature of 60°, continually passing over the head. This treatment is said to have proved successful, often in one or two weeks.

---

☞ Our present issue will doubtless fail to reach all its readers in the State, before the second Wednesday in April, inst., the day for convening of the Medical Association of Georgia. It is not important, however, that it should, since, in our March No. attention was called to the fact. We regret the omission of other journals in the State to mention the time of meeting or in any way allude to the meeting. We presume, however, the April No. of their journal will carry out a notice, and hope it will reach their readers in time.

☞ The six-parts—300 pages each—of an Epitome of Braithwaites' Retrospect of Practical Medicine and Surgery, by Walter S. Wells, M. D., and published by C. T. Evans, New York, have been received. We know of no work more useful to the practical physician than this. It is a compilation of twenty years—forty volumes—of Braithwaite's Retrospect, in which the cream of that valuable publication is collected. The work may be had of booksellers in this city. The author proposes to continue his labors " under the title of Summary of Medical Sciences," as will be seen in advertisement.

